# Corrosion Performance of Atmospheric Corrosion Resistant Steel Bridges in the Current Climate: A Performance Review

**DOI:** 10.3390/ma18153510

**Published:** 2025-07-26

**Authors:** Nafiseh Ebrahimi, Melina Roshanfar, Mojtaba Momeni, Olga Naboka

**Affiliations:** 1 Construction Research Center, National Research Council of Canada, 1200 Montreal Rd., Ottawa, ON K1A 0R6, Canada; mrosh022@uottawa.ca (M.R.); olga.naboka@nrc-cnrc.gc.ca (O.N.); 2 EC Energy, London, ON N6G 5N7, Canada; mojtaba.momeni@ecenergy.ca

**Keywords:** weathering steel, bridge corrosion, protective patina, alloying elements, climate resilience, maintenance strategies

## Abstract

Weathering steel (WS) is widely used in bridge construction due to its high corrosion resistance, durability, and low maintenance requirements. This paper reviews the performance of WS bridges in Canadian climates, focusing on the formation of protective patina, influencing factors, and long-term maintenance strategies. The protective patina, composed of stable iron oxyhydroxides, develops over time under favorable wet–dry cycles but can be disrupted by environmental aggressors such as chlorides, sulfur dioxide, and prolonged moisture exposure. Key alloying elements like Cu, Cr, Ni, and Nb enhance corrosion resistance, while design considerations—such as drainage optimization and avoidance of crevices—are critical for performance. The study highlights the vulnerability of WS bridges to microenvironments, including de-icing salt exposure, coastal humidity, and debris accumulation. Regular inspections and maintenance, such as debris removal, drainage system upkeep, and targeted cleaning, are essential to mitigate corrosion risks. Climate change exacerbates challenges, with rising temperatures, altered precipitation patterns, and ocean acidification accelerating corrosion in coastal regions. Future research directions include optimizing WS compositions with advanced alloys (e.g., rare earth elements) and integrating climate-resilient design practices. This review highlights the need for a holistic approach combining material science, proactive maintenance, and adaptive design to ensure the longevity of WS bridges in evolving environmental conditions.

## 1. Introduction

The term “weathering steel” (low-alloyed steels) is used for mild steels containing less than 0.2% carbon, containing other elements such as copper (Cu), chromium (Cr), nickel (Ni), phosphorus (P), silicon (Si), and manganese (Mn). The added elements could be in a range of 1–5 wt.% [1]. Weathering steels are known for their high corrosion resistance (2–8 times higher than plain carbon steel (CS)), high strength, excellent toughness, and self-healing properties [1,2,3]. Some WS applications include bridges, load-bearing structures, transmission towers, buildings, and decorative features. When exposed to the atmosphere, WS surfaces are protected from further corrosion by forming a patina or rust protective layer. Its color varies depending on environmental conditions, such as atmosphere corrosivity, the type of steel (components), and the exposure time, and this is why WS is used for artistic purposes [2,3]. The protective rust layer may take 6–8 years to form in less aggressive environments, while in more corrosive environments, it may take 4–6 years [4]. After forming a protective layer, steady-state corrosion is at a rate of 5–6 µm/year [2,5].

In 1933, the first commercially available WS was produced under the name Cor-Ten, which refers to improved corrosion resistance (Cor) and mechanical properties (Ten) over plain carbon steel [2,6]. In 1941, ASTM A-242 [7] was published as the first standard for WS. In 1968, WS was classified into two categories (high P content (0.07–0.15 wt.%) and low P content (<0.04 wt.%)). Since the second category of WS has a low P content, it is less resistant to atmospheric corrosion. Thus, ASTM A-588 [8] was replaced for WSs with a low P content [2]. Table 1 shows the chemical compositions of WS in ASTM A-242 and ASTM A-588 [2]. For new construction, ASTM A-709 [9] was published in 1975 for WS, and 50W, HPS 50W, HPS 70W, and HPS 100W are strongly recommended (US). Nowadays, steelmaking companies in diverse countries manufacture their own WS under various brand names, such as Cor-Ten (US steel, Pittsburgh, PA, USA), COR-TEN (Thyssen Krupp, Essen, Germany), Mayari (Bethlehem Steel, Bethlehem, PA, USA), Atmofixs (Nové Město, Czech Republic), and ENSACOR (Panama, Spain) [2]. Table 2 lists the most used weathering steels around the world and compares their designation in different regions. The presented comparison shows that most of the currently used weathering steels across the world are developed based on ASTM A-242 and ASTM A-588 alloys.

## 2. Mechanism of Patina Formation

As one of the main components of the protective rust layer, Misawa characterized fine particles of amorphous ferric oxyhydroxide [FeO_x_(OH)_3−2x_] and considered them as a feroxyhyte (γ-FeOOH). Misawa proposed that after the dissolution of iron ions, ions hydrolyze, and FeOH is produced. Then, FeOH is oxidized and precipitated as γ-FeOOH (lepidocrocite). Subsequently, γ-FeOOH is dissolved and precipitated again as δ-FeOOH (feroxyhyte). Finally, δ-FeOOH is transferred to α-FeOOH (geothite) through solid-state transformation [1,2].

Stratmann suggested that the mechanism could be divided into three stages. Stage 1 (Wetting of Dry Surface): During this stage, the iron anodic dissolution is balanced by lepidocrocite cathodic reduction in the rust layer. Stage 2 (Wet Surface): Oxygen was reduced in the oxide. Stage 3 (Drying out of the Surface): During the drying phase, the patina layer experiences several changes. First, the electrolyte film thins on the inner surface of the rust layer, which is rapid diffusion-limited O_2_ reduction. Then, the stage would have a very high corrosion rate. In addition, the Fe^2+^ intermediate (γ-FeOHOH) formed in stage 1 can also undergo reoxidation to γ-FeOOH. Subsequently, γ-FeOOH transferred to goethite. These changes in the patina layer are complete when the electrolyte has been completely consumed [1,2].

**Figure 1:**
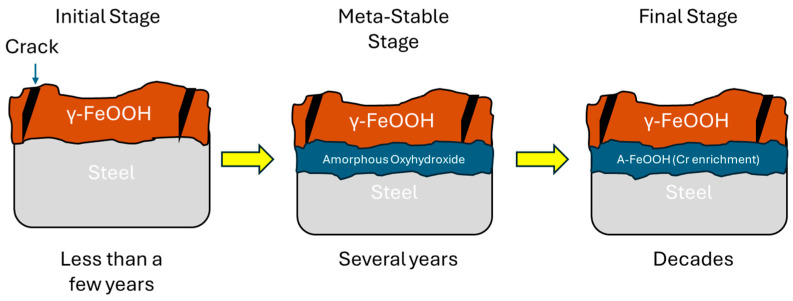
The schematic model of Yamashita for rusting [5,21].

Later, Yamashita proposed another mechanism, which is shown in Figure 1. Yamashita’s mechanism describes an outer layer rich in lepidocrocite and an inner layer with substantial goethite, differing in particle packing. Additionally, the inner layer comprises fine particles that are closely packed, whereas the outer layer is composed of loosely aggregated components. A significant Cr concentration was observed only in the inner layer of Cr-substituted goethite nanoparticles (Cr-FG) [1,2].

**Figure 2:**
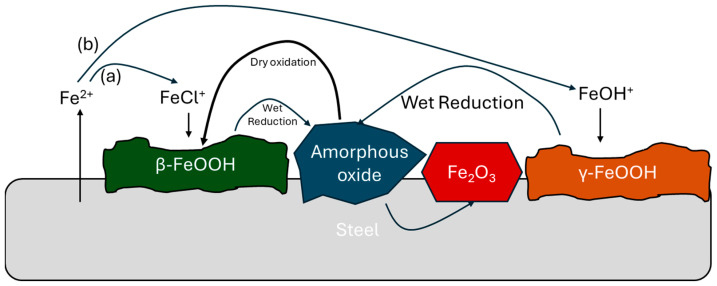
The rusting mechanism in the presence of NaCl in (a) high Cl ions and (b) low Cl ion concentration [2].

Figure 2 shows the rusting mechanism if NaCl is present in the environment. In saline environments (like marine), akaganeite (β-FeOOH) forms on WS, leading to a different rusting mechanism. Increased NaCl concentration accelerates steel corrosion and leads to a higher proportion of akaganeite over 0.05 wt.% NaCl. However, lepidocrocite can be formed from Fe(OH)_2_ at a low corrosion rate when Cl^−^ concentration is low (0–0.30% Cl^−^) [2].

Using Protective Ability Indices (PAIs), it is possible to evaluate the effectiveness of the rust layer. PAI uses the goethite to lepidocrocite (α/γ) mass ratio (from XRD) as a function of exposure time. As exposure time increases, the ratio increases proportionally. α/γ should be more than 2 to form a protective rust layer. Therefore, the α/γ ratio can be considered as an indicator. However, this increasing α/γ trend with exposure time does not hold in marine atmospheres. Thus, γ* was defined (as shown in Equation (1)) as the ratio of goethite to lepidocrocite + akaganeite + spinel (S, magnetite (Fe_3_O_4_) and/or maghemite (γ-Fe_2_O_3_)) as follows [2,5]:

(1) $$\frac{\alpha }{\gamma^{*}}=\frac{\alpha }{\gamma +\beta +S}$$

**Figure 3:**
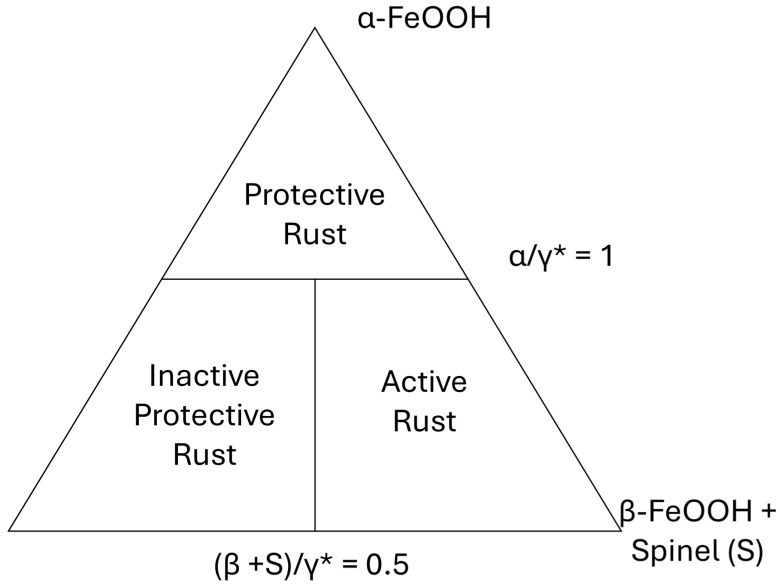
Ternary diagram of rust layer composition [5].

Steel corrosion rates exceed the threshold (α/γ*) if the rate of corrosion is higher than 10 μm/year. Figure 3 depicts a diagram for assessing the ability of rust to protect steel based on the α/γ* in various environmental conditions.

Therefore, the diagram and the formula can be used as follows:

(a) Protective Rust: When α/γ* is greater than 1, a corrosion rate of >10 µm/year would not be observed.

(b) When α/γ* is lower than 1, the corrosion rate should be determined by (β + S)/γ*. It could be replaced by other terms if the corrosion rate is more than or less than 10 µm/year ((β + S)/γ*, whereby (β + S)/γ* > 0.50 (active non-protective rust) and (β + S)/γ* < 0.50 (inactive protective rust)) [5]. Other ratios such as α*/γ* were also proposed. If magnetite shows relatively high stability, the following ratio should be applied:

(2) $$\frac{\alpha^{*}}{\gamma^{*}}=\frac{\alpha +S}{\gamma +\beta }$$

where α is the fraction of α-FeOOH, S is the fraction of the spinel phase, γ is the fraction of γ-FeOOH, and β is the fraction of β-FeOOH.

Also, thickness loss of WS can be calculated as follows:

(3) $$D=\frac{W}{\rho A}\times 10^{4}$$

where D is thickness loss (μm), W is weight loss (g), ρ is the density of steel (7.85 g/cm^3^), and A is the sample exposed area (cm^2^) [22].

## 3. Alloying Elements

WS is distinguished by its distinctive properties as a result of the addition of alloying elements. In other words, it is believed that the outstanding corrosion resistance may be attributed to the addition of a limited amount of alloying elements to form a dense and well-adhered corrosion product film (CPF), which primarily consists of α-FeOOH, β-FeOOH, γ-FeOOH, Fe_3_O_4_, and amorphous phases. In corrosive environments, such as marine atmospheres, in which corrosive ions, high humidity, high temperature, and heavy rainfall are present, the layer may be destroyed, resulting in a shorter service life for WS [3]. As a result, it is essential to investigate the effects of each element to produce a WS with improved functionality [6].

### 3.1. Phosphorus

Phosphorus has a negative impact on mechanical properties, as previously stated. However, complexing with Cu may provide anticorrosive properties. The use of P is therefore limited, and it should not exceed 0.1 wt.% [1]. Phosphorus (P) plays a complex yet significant role in enhancing the corrosion resistance of weathering steels by influencing the formation, stability, and protective qualities of the patina. Its contributions are both chemical and structural, working synergistically with other alloying elements to create a dense, adherent rust layer that slows further degradation. It is reported that phosphorus contributes to the patina formation via (a) promotion of stable rust phase [23,24], (b) formation of protective phosphide layers [25], and (c) long-term stability, where after long-term (+10 years), it forms a higher proportion of crystalline stable phases like α-FeOOH [26].

### 3.2. Copper

Increasing the Cu content in steel improves corrosion resistance and steel service time, and it has a positive impact on weathering steel’s properties. The addition of 0.04 wt.% of Cu enhances anticorrosive behavior in the atmosphere, but additions above 0.25 wt.% show little improvement [1,5]. It is important to note that Cu plays an essential role in achieving higher corrosion resistance.

### 3.3. Chromium

In marine environments, copper addition does not improve corrosion resistance, as compared to rural and industrial environments. Therefore, additional chromium was suggested to solve the issue. Previous studies have shown that Cr has a significant effect on the atmospheric corrosion resistance of WS. A minimum of 0.1 wt.% of copper must, however, be present in the steel composition to achieve a significant effect on the improvement of atmospheric corrosion [1,5]. The corrosion resistance of WS with Cu (0.30 wt.%) and Cr (1.53 wt.%) has been shown to be better than that of plain carbon steel [1,5]. It is worth noting that Cr generates spinel Fe_2_CrO_4_ as a protective oxide, which increases the amount of α-FeOOH and improves the protection [27]. Cr will be deposited in the inner rust later and form a relatively dense layer. In addition to improving FeOOH formation, Cr addition also increases Cr_2_O_3_ formation in the rust layer, resulting in increased corrosion resistance [6].

### 3.4. Nickel

Weathering steel can be improved in terms of corrosion resistance by adding nickel. For example, 1 wt.% of Ni and a small percentage of Cu could improve corrosion resistance in marine and industrial environments [1]. Higher Ni content can reduce the active dissolution rate of WS. Therefore, acidification would be lower at the steel–rust interface, and the hydrolysis of metal ions would be more straightforward. This would stabilize Fe (II, III) oxides, thereby promoting alkalization at the corrosion interface. As Ni is present in WSs, it forms spinel Fe_2_NiO_4_, which increases the proportion of α-FeOOH, improving the effectiveness of CPF protection [27]. For example, a WS with 3 wt.% of Ni can be employed without a protective paint coating in an atmosphere with a Cl deposition rate of 20 mg Cl^−^/m^2^/day [5].

### 3.5. Calcium

Calcium (Ca) addition is intended to raise the pH at the corrosion interface, promote iron oxyhydroxide stabilization, alter the ion-exchange properties of colloidal corrosion products, and maintain a passive state at bare areas on a steel surface caused by irregularities in the rust layer [5].

### 3.6. Niobium

Niobium (Nb) is widely used as a microalloying element in high-strength, low-alloyed steels. Nb has a high tendency to carbon (C) and nitrogen (N), and their precipitants can improve steel properties, such as strength, ductility, toughness, and mechanical properties. Interestingly, Nb-containing steels exhibit greater corrosion resistance than steels without Nb. This could be because of NbC nano-precipitates. These nano-precipitates can also trap hydrogen and hinder pitting corrosion. As a result, hydrogen embrittlement sensitivity decreases, and anti-corrosivity increases [3,28]. A limited number of pits were observed in steels containing Nb. The presence of Nb alleviates acidification and further reduces the corrosion rate of WSs [27]. Therefore, Nb promotes the formation of a protective layer, and increasing Nb improves the properties of the rust layer [29,30]. Incorporating Nb with P and S products results in a substantial increase in iron oxyhydroxide concentrations. This causes the film layer to become thicker and more compact. Rust formed on the steel is certainly dense enough to provide protection. A corrosion product may also form on Nb steels when an Nb-containing compound, probably niobium oxide, is present and the steel surface is enriched due to increased Nb concentration. Thus, the improved film significantly reduces the corrosion rate of Nb steels [29].

### 3.7. Other Elements

**Figure 4:**
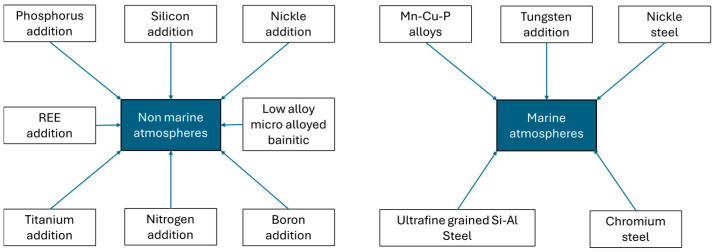
New WSs according to studies.

Vanadium (V), titanium (Ti), and antimony (Sb) are the other microalloying elements that have shown promising results and promote protective layer formation [3,27,28]. In addition, manganese, particularly manganese sulfide (MnS), can provide initiation sites for pitting corrosion in both carbon steel and stainless steel. The corrosion resistance of steel is also adversely affected by SiO_2_, CaS, and Ca-Al-Mg-O [27]. It was reported that boron (B) addition enhances the mechanical properties of WSs. Also, B addition reduces a WS’s pearlite content, which negatively affects corrosion. Therefore, it can improve corrosion resistance. A small amount of rare earth elements (RE, 0.01 = 0.03 wt.%) also increases protective layer formation, provides a more compact layer, and improves mechanical properties [1]. Figure 4 shows new weathering steels studied in previous studies [5].

## 4. Effective Parameters of Corrosion Performance

Different parameters affect the formation of rust layers, for example, wet–dry cycling, the length of wetness time, humidity, Cl ions, sulfur dioxide, dust, exposure time, and the location of a WS bridge [6]. Each parameter is discussed in detail below.

### 4.1. Wet–Dry Cycle or Time of Wetness

Weathering steel is greatly affected by the wet–dry cycle or the time of wetness, and a reasonable wet–dry cycle is essential for forming a dense, adherent rust layer. There is a linear relationship between the thickness of the rust layer and the rate of substrate corrosion as exposure time increases. To remove dust, debris, and salts from the WS, wet time is required, and fast drying is necessary to remove the moisture [1,2]. Generally, extended periods of wetness are found in areas close to the ocean or in areas with high rainfall, such as the Pacific West of Canada. In such environments, uncoated WS might not be suitable [31]. There is a threshold for classifying locations according to the time of wetness. The US guide considers an area with a wet period exceeding 5500 h per year (i.e., approximately 60% of the year) a severe location (T5). In such areas, WS behaves similarly to plain carbon steel [32]. Australian WS and UK guide also considered 60% of the year as a threshold. Table 3 shows the category of environments according to the time of wetness. Australian WS guide for the uncoated WSs that are in a region where the time of wetness is greater than 60% and up to 70%, provided the following restrictions:

- At any time of the year, the bridge must not be shaded by permanent obstructions, such as surrounding hills between the hours of 9 a.m. and 3 p.m.
- There must be an unobstructed flow of air over the steelwork of the bridges.
- Within three meters of the bridge steelwork, no vegetation must be higher than grass.

In addition to the macro-environment (the surrounding atmosphere), some important factors called micro-environments should also be considered. When parts of a WS bridge are immersed in water (water crossing bridges), buried in soil, or covered with vegetation, they can act similarly to extended wetness time and accelerate corrosion [2,31,32,33]. Flooding that occurs repeatedly or over a prolonged period of time results in excessively wet conditions. Despite this, floods also frequently cause debris to be trapped on superstructures, resulting in trapped moisture on them. Due to the moisture trapped in the debris, corrosion can be greatly accelerated over a long period of time. It is recommended that the frequency of flooding that may occur at different elevations be considered, and plans should be developed to anticipate that debris removal may be required after floods. Due to their protection from the sun, the downward parts of bridges usually remain wet for a longer period than the upward parts [2]. When exposed to excessive wet, such parts develop non-protective rust layers [5]. Using a drainage system can help extend the lifetime of WS bridges. For instance, a drainage system is mandatory to prevent water, wet soil, or debris from accumulating on lower flanges [5].

### 4.2. Humidity

There is a direct connection between humidity and wetness time. According to the US guide [31], a location is considered humid if the relative humidity exceeds 75% for eight or more months of the year, as quantified by the NOAA CAUS dataset. Many factors may contribute to humidity, including the general climate, surface water, and vegetation growing against the bridge. Considering the amount of time that the relative humidity is high, the coastal environment is considered a relatively humid environment. Datasets from organizations such as the National Oceanic and Atmospheric Administration (NOAA) are required to classify other locations; for example, the Climate Atlas of the United States (CAUS), published by NOAA, is commonly used [31].

Similarly to wet–dry cycles, it is recommended to provide fully enclosed rain drainage systems that keep the steel dry enough. To minimize the effects of wind-driven spray on steel, the drainage system must expel water sufficiently far away from the steel. A deck overhang is a good drainage system [31]. Based on the reported climate change data, the humidity increases by 5–10% if the Earth’s temperature rises by 2.5–6.5 °C. Thus, it is estimated that a 100-year design life structure will lose 15 years of service life on average, and climate change may contribute to the acceleration of infrastructure deterioration, especially along the coast [4].

### 4.3. Chloride Ions (Cl^−^)

The major aggressive species for WS is Cl ions. It can penetrate the rust layer and promote the formation of akageneite (β-FeOOH), and non-protective rust is formed due to the tunnel structure of akageneite [1,34]. In other words, anion-selective permeability was enhanced in a high-chlorine environment in a marine environment, accelerating corrosion [35]. A brown or orange patina results from the penetration of Cl, and mass loss and total metal release increase linearly with time and concentration, with no signs of stabilization in the ranges examined [5]. Generally, due to the presence of chlorides in marine environments, corrosion rates are higher than those in urban environments [21]. In addition, a WS bridge can be exposed to Cl ions if it is in contact with de-icing salts or salted water. Different countries have proposed different threshold amounts for Cl ions. Initially, a critical chlorine level of 3 mg/m^2^/day (0.05 mg NaCl/dm^2^/day) was established for airborne salinity in Japan, where corrosion rates for WS are limited to 6 µm/year. However, this level has been increased to 6 mg/m^2^/day, and there is even discussion of a range of 6–12 mg/m^2^/day based on service conditions. The Department of Transport of the United Kingdom had considered Cl levels exceeding 10 mg/m^2^/day. However, they increased it up to 300 mg Cl/m^2^/day. In the US, it is recommended to avoid >50 mg Cl/m^2^/day [1,2,5]. Climate change datasets indicate that Cl-induced corrosion may occur 2–18% earlier than anticipated in coastal areas, and structure failure may occur 3–31% earlier [4].

De-icing materials consist of Cl ions. Thus, locations frequently exposed to de-icing materials are considered severe in terms of corrosion, such as rain-sheltered surfaces, where chlorides accumulate and are never washed away [1]. As stated in the US guide, bridge roads that are in contact with de-icing salts or marine salts are more likely to corrode. According to European and United Kingdom guidelines, the underside (superstructure) of the overpassing bridge is exposed to de-icing salt spray from the road below, which impacts the microclimate underneath and may increase corrosion rates. However, Cl concentrations at overpassing structures are acceptable for most conventional intersection structures that do not have retaining walls adjacent to the road below [33]. Also, the UK guide mentioned that leaking expansion joints caused salt-laden run-off to flow directly over the steel and salt spray from roads under wide bridges where tunnel-like conditions are created. Spraying occurs on the underside of bridges above highways since the spray envelope from vehicles using the road below can extend as high as 7.50 m [2]. However, salt spray is unlikely to cause a problem for weathering steel composites over bridges at 5.3 sm headroom [32]. Australian guidelines emphasize that high concentrations of Cl ions are harmful to the formation of patina. WS could contact Cl ions through breaking waves at sea, on the shoreline, or in salt fogs. Weathering steel bridges may also be affected by de-icing salts used on roads both over and beneath them. Because of the limited application of de-icing, it, however, does not cause any problems in Australia [36].

### 4.4. Sulfur Dioxide (SO_2_)

Sulfur dioxide is also an aggressive species for WS corrosion. To keep the steady-state corrosion rate of 6 μm/year, the SO_2_ amount should be less than 20 mg SO_2_/m^2^/day. When this threshold is exceeded, WS corrosion is accelerated, as SO_2_ causes intense acidification of the aqueous layer present on WS during corrosion, resulting in dissolution and inhibiting precipitation [1,2,34]. In general, SO_2_-polluted atmospheres are found at industrial sites, especially those that use fossil fuels [2]. In rural–urban, industrial, and marine atmospheres, SO_2_ pollution can be simulated using the Cebelcor, Kesternich, and Prohesion cyclic tests, respectively. If SO_2_ concentrations in the atmosphere are very high, many situations fall into the C4–C5 categories of atmospheric corrosion (ISO 9223 [37]). SO_2_ causes the development of a non-adherent patina, especially around joint parts and in and around crevices [5]. European guidelines state that SO_2_ levels higher than 80 mg SO_2_/m^2^/day are considered indicative of a highly polluted industrial atmosphere. Due to the environmental regulations in Europe, the level of SO_2_ was decreased in the environment; therefore, the threshold was increased to 45 μg/m^3^ [33]. In accordance with ISO 9223, the UK and Australia define the threshold as SO_2_ levels exceeding 250 μg/m^3^. This level, however, is extremely rare [32,36]. The United Kingdom Department of Transport has specified that uncoated WS shall not be used when SO_2_ levels exceed 168 mg/m^2^/day. However, in 2001, new critical levels of 200 mg/m^2^/day were established. According to U.S. government guidelines, conditions that should be avoided using a WS are those in which SO_2_ exceeds 168 mg of SO_2_/m^2^/day. The guidelines for using uncoated weathering steel in the U.S. consider 250 μg/m^3^ as the maximum allowable sulfate concentration, and the U.S. Environmental Protection Agency limits sulfur dioxide emissions to 200 μg/m^3^ [31].

As mentioned, due to environmental regulations, SO_2_ levels in the atmosphere have significantly decreased in recent decades. Additionally, the sulfur content in steel decreased dramatically, which is a particularly harmful chemical element from the perspective of atmospheric corrosion. The decrease between 1966 and 2019 in thickness loss due to corrosion is primarily due to changes in environmental data, namely the concentration of SO_2_ in the atmosphere [21].

### 4.5. Dust and Debris

Corrosion could be significantly higher if the WS were covered with solid particles (dust, dirt, soot) since moisture and salts were retained for longer periods on the surface [1,31].

### 4.6. Environment and Location

There are four types of environments: rural, urban, industrial, and marine. In all WS guidelines, rural and urban environments are considered benign in terms of macro-environment, and other parameters, such as de-icing agents, are regarded as micro-environments. In the industrial environment, sulfur dioxide would be of particular concern (although it is not a concern anymore). Marine/coastal environments incorporate humidity and Cl concerns and are the most aggressive environments. According to studies, a patina developed in the first four years of exposure to an industrial atmosphere is more protective than a patina formed in a marine environment [1]. U.S. guidelines have, however, highlighted bridges in rail yards as an example of a potentially severe and highly specific high sulfate environment [31]. According to the U.S. guidelines, the uncoated WS is a suitable choice for crossing railroad tracks, railroad bridges, and pedestrian bridges. However, weathering steels should not be immersed in water, in contact with soil, or covered in vegetation [31,36].

The marine/coastal atmosphere is defined differently by different guidelines. It is considered a coastal environment in the U.S. if a bridge is less than two miles (3.22 km) from the coast and has an average relative humidity of greater than or equal to 75 percent for eight or more months during a year [31]. According to German guidelines, a minimum distance of 500 m must be maintained outside of permanent fog. French guidelines require a distance of 2 km from the North Sea, the English Channel, and the Atlantic Ocean, but only one kilometer from the Mediterranean Sea. According to UK guidelines, a WS bridge may not be constructed up to 2 km from the coast. However, European guidelines generally recommend 1–2 km from a coastline [32,33]. According to Australian guidelines, WS bridges should not be located within 2 km of an open coast. Additionally, the corrosion rate should not exceed 50 μm/yr in the first year. Depending on the strength and direction of prevailing winds, ocean waves, coastal surf conditions, topography, obstructions to wind flow, and the level of shelter near the site, the distance could be extended up to 40 km [36].

### 4.7. Bridge Design

Several general design factors should be considered. Ideally, complex designs should be avoided, and good drainage should be provided [5]. Structures must be free of interstices, crevices, cavities, and other areas where moisture can collect, as corrosion may occur there without the formation of a protective patina [1,2,33]. Also, water should not continuously flow over or pond on the steel surfaces, which could be the most important design recommendation [31]. Corrosion can occur in bolted joints due to moisture accumulation resulting from condensation or rain [5]. For crossings over water, UK guidelines recommend a minimum headroom of 2.5 m [32,36]. Due to their long-term wetness, leaking expansion joints present a major concern [32,36]. Corrosion rates are higher on horizontal, sloping, and downward-facing surfaces in sheltered interior locations than on comparable upward-facing surfaces. Surfaces that are vertical are less likely to experience pitting and corrosion. The salt and Cl accumulations are also greater in partially enclosed boxed locations and regions where water accumulates and stagnates [5].

Surfaces that are protected from the sun and rain, known as sheltered surfaces, develop loose and poorly compacted rust. In this regard, the rate of corrosion would be higher than other parts of a bridge due to the longer moisture exposure and the lack of pollutant wash-off by rain [2,31,32,36]. It is crucial to consider the degree of sheltering and the orientation of the surface [5]. Tunnel-like conditions occur when a narrow road passes beneath a wide one. In this case, the de-icing salts and moisture will deposit for a more extended period of time, and the corrosion rate will be high [2,31,32]. The tunnel-like effect is considered in Australia, where headroom is less than 5.3 m [36].

## 5. Inspection

It is important to acknowledge that weathering steel requires maintenance and can be susceptible to significant corrosion due to design and construction errors, environmental conditions (such as topography), and the use of de-icing salt. Therefore, it is necessary to conduct regular inspections of the steel to monitor its condition [36,38]. Several parts of a bridge, including supports, expansion joints, and beams, are more prone to corrosion due to their tendency to accumulate moisture. This prevents the formation of a protective rust layer, leading to severe corrosion and a compromise in structural integrity [38]. The bridge design should ensure that all components are readily accessible for thorough inspections [31,33,36,39].

Weathering steel inspections generally do not differ significantly from those of carbon steel. It is advisable to begin the initial periodic assessment approximately two years after the weathering steel bridge’s completion. Subsequently, routine inspections should occur consistently, with a minimum frequency of two years. These general inspections should focus on areas where developing a protective rust layer is challenging. These areas include the visual condition of the rust layer on the weathering steel surface, expansion joints, drainage systems, and joint corrosion [31,33,36].

**Figure 5:**
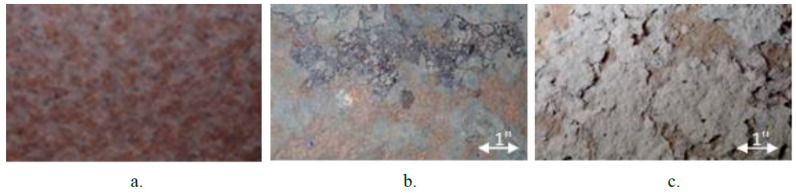
The surface of a WS is (**a**) a rough, freckled surface, (**b**) a smooth surface that was easily crushed by tapping, and (**c**) a course surface with an exfoliating rust layer [31].

Inspections can be conducted by assessing the color and appearance of the surface. The color and texture of the rust layer provide valuable insights into the protectiveness of the oxide formed on the weathering steel. It is important to consider various rust layer characteristics, such as color, texture, roughness, and thickness. A protective rust layer on the surface serves as a reliable performance indicator. A firmly attached, finely textured rust patina signifies that corrosion is progressing at an acceptable rate. On the other hand, coarse, stratified rust layers and peeling are indicative of unsatisfactory performance [36]. In most cases, there is no need to be concerned about particles with a diameter of 1/8″ or less. Particles of this size create relatively smooth surfaces, resulting in a range of appearances. However, granular rust flakes exceeding ¼″ in diameter may suggest the presence of a non-protective patina. Additionally, sheet-like rust layers are clear evidence of a non-protective patina, as demonstrated in Figure 5 [31].

**Figure 6:**
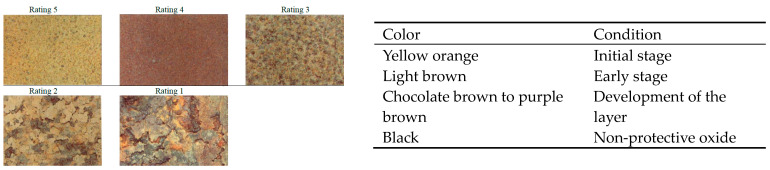
Rust layer color in different conditions [38,40].

In general, rust layer appearances are typically rated on a scale from 1 to 5, with 5 indicating the most stable and 1 representing the most unstable condition. Table 4 illustrates the relationship between rust layer thickness and appearance, while Figure 6 showcases the surface color of weathering steel under various conditions. Following the initial exposure, the protective rust coating is expected to transition from yellow-orange to light brown and eventually develop a chocolate-brown color [31,36]. However, in typical U.S. environments, non-protective oxides tend to appear dull gray or black. It is of utmost importance for an inspector to comprehensively understand the various colors, textures, and overall appearances of the rust patina when exposed to different environmental conditions.

**Figure 7:**
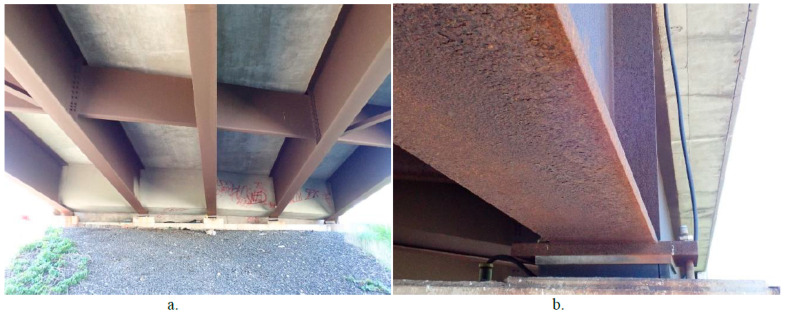
The surfaces of the WS differ in appearance when viewed from (**a**) a distance and (**b**) a close distance [31].

Note that the visual appearance of the weathering steel surface can be misleading [31,36,38]. In general, a poorly performing surface is typically characterized by granular or flaky appearances. While the patina may initially appear acceptable from a distance, closer inspection may reveal large flakes, potentially indicating less than satisfactory performance. As depicted in Figure 7, weathering steel surfaces exhibit differing appearances when viewed up close versus from a distance. Therefore, during the examination of the surface rust layer, it is essential to assess the adherence of the rust’s oxide film to the underlying steel substrate by tapping it with a hammer and vigorously brushing it with a steel wire. Additionally, the oxide layer can be readily removed using a mechanical wire brush attached to a drill or a similar tool. This assessment aids in accurately determining the integrity of the oxide film attached to the steel substrate below or if it is only present in the form of particles, scales, or layers. Tape tests provide a means to assess the size and density of corrosion products. This process involves applying tape that meets ASTM D3359 [41] requirements (typically clear packaging tape) to the steel surface. Subsequently, the size and spatial density of the corrosion particles adhering to the tape are measured [31,36]. This test is relatively easy to perform, requiring minimal training and readily available supplies. While tape sample data may not be as straightforward as using ultrasonic thickness data, it can offer valuable insights into potential corrosion issues [38]. However, it is important to note that certain tests, such as wire brushing or surface preparation for ultrasonic examinations, can alter the appearance of the protective layer. It may take some time for the surface to return to its original state [36].

During the initial decade of exposure, the corrosion rate typically remains elevated before stabilizing at a lower level. As a result, it is crucial to gather corrosion data for a minimum of 20 years unless substantial unintended structural degradation is observed within this timeframe. If the corrosion rate exceeds the level upon which the initial corrosion allowance was based, it becomes imperative to investigate the root causes of corrosion and implement remedial actions for affected areas [38]. It is worth noting that the timeframe required for the protective layer to fully develop is influenced by environmental factors. For example, in New Zealand, it has been observed that under optimal conditions, it may take up to 8 years for the protective patina to mature. However, in regions with prolonged periods of wetness, this period can extend to 16 years or even longer [36]. In a “mild” exposed environment, such as Ireland, corrosion rates are expected to be less than 10 μm per year following the first few years of exposure [39].

### 5.1. Inspection Procedure

**Figure 8:**
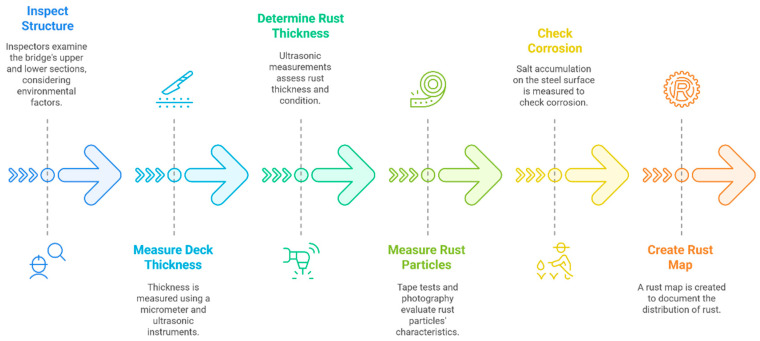
Bridge inspection procedure.

A systematic inspection procedure is essential for evaluating the performance and integrity of the weathering steel in bridge infrastructures, as shown in Figure 8. The following steps outline the key inspection activities [31,33,36,38,39,42]:

**Inspect the Structure:** Inspectors assess the bridge’s upper and lower sections, considering its environmental exposure and location. A close-range evaluation (within one meter) is necessary to distinguish between protective and non-protective rust coatings. Environmental factors such as humidity, pollutants, and Cl deposition should also be considered.

**Measure Deck Thickness:** The thickness of the bridge deck is measured using micrometers and ultrasonic instruments, particularly on piers and abutments. Thickness reduction over time helps assess the structural integrity of the bridge. Standard guidelines suggest conducting corrosion rate measurements every six years in a C3 environment and every twelve years in a C2 environment.

**Determine Rust Thickness:** Ultrasonic measurements are employed to determine the rust layer’s thickness and overall condition. A rust thickness of less than 400 μm is generally considered acceptable, whereas values exceeding this threshold indicate potential durability concerns. Long-term data collection (at least 20 years) is recommended unless unexpected structural deterioration is observed.

**Measure Rust Particles:** Rust particle distribution and characteristics are assessed through tape tests, close-up photography, and imaging techniques. Factors such as particle amount, size, color, uniformity, and surface unevenness are documented. For fine crack detection, magnetic particle inspection can be used alongside ultrasonic testing to enhance precision.

**Check Corrosion:** Salt accumulation on the steel surface is measured as an indicator of corrosion susceptibility. Elevated salt levels may accelerate corrosion rates, necessitating further protective measures.

**Create a Rust Map:** A rust distribution map is generated to document the spatial variations in corrosion across the bridge structure. This record helps in tracking corrosion progression and planning maintenance interventions.

This inspection procedure ensures a comprehensive evaluation of weathering steel performance in bridge applications, contributing to long-term structural reliability.

### 5.2. Checklist for Inspectors

**Figure 9:**
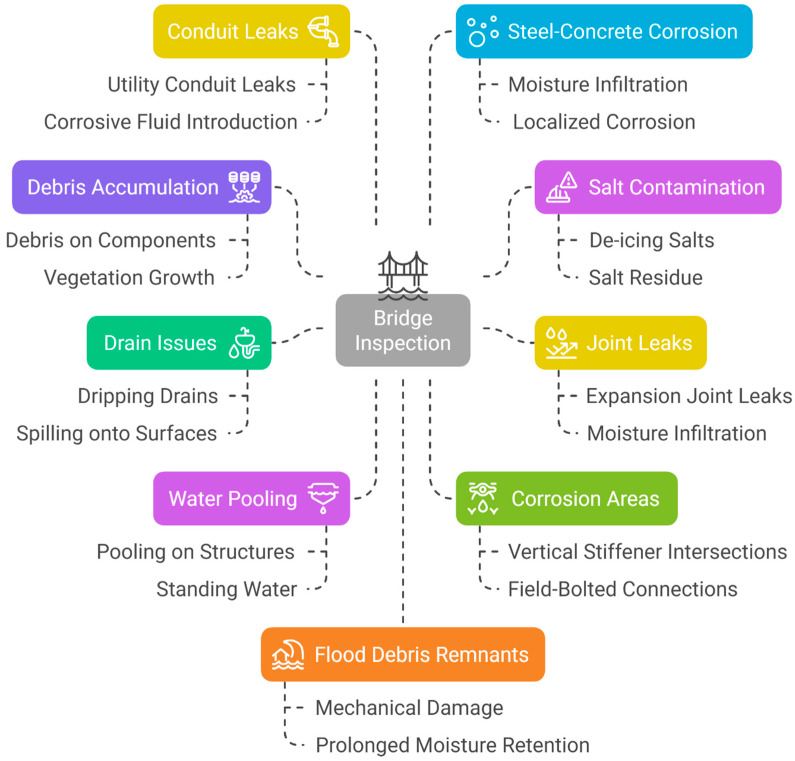
Regular inspection checklist.

Regular maintenance inspections (Figure 9) are crucial to ensuring the longevity and structural integrity of weathering steel bridges. The following key issues should be assessed during inspections:

**Debris Accumulation:** Inspect for the presence of debris or vegetation around bridge components, particularly in areas exposed to frequent moisture. Accumulated debris can retain moisture, accelerating localized corrosion.

**Salt Contamination:** Check for salt residue on steel surfaces, which can increase the risk of Cl-induced corrosion, especially in regions where de-icing salts are used.

**Joint Leaks:** Identify any signs of leaking expansion joints. Leaks can introduce moisture into structural elements, leading to accelerated deterioration.

**Drain Issues:** Assess surface runoff drains for signs of dripping or spilling onto steel surfaces or substructures, as improper drainage can contribute to corrosion and water accumulation.

**Water Pooling:** Determine if water is pooling and persisting on steel structures, bearings, or substructures. Prolonged exposure to standing water can compromise protective patina formation and lead to accelerated corrosion.

**Corrosion Areas:** Inspect for obstructions or corrosion at critical locations, such as where vertical stiffeners intersect with the bottom flange or at field-bolted connections. These areas are particularly vulnerable to corrosion due to water entrapment and differential aeration effects.

**Conduit Leaks:** Examine utility conduits for potential leaks onto steel surfaces or substructures. Leaking fluids can introduce corrosive contaminants that degrade steel components over time.

**Steel–Concrete Corrosion:** Evaluate corrosion at the interfaces between steel and concrete. Moisture infiltration at these junctions can lead to localized corrosion and reduced structural integrity.

**Flood Debris Remnants:** Inspect for remnants of debris from waterway flooding. Flood-induced debris can cause mechanical damage and increase the risk of prolonged moisture retention on steel surfaces.

By systematically addressing these maintenance issues, inspectors can mitigate deterioration and extend the service life of weathering steel bridges [31,33,36,40].

### 5.3. Vulnerable Members of Bridges

It is important to consider that the performance of weathering steel bridges is heavily impacted by the microclimates specific to each environment. For example, a weathering steel structure in a marine environment may thrive, while one in a rural environment might deteriorate due to micro-environmental variations [40,43,44]. Therefore, it is crucial to highlight that the performance of weathering steel bridges can be significantly affected by microenvironments (bridge-specific).

Certain bridge components are particularly vulnerable to corrosion due to their exposure to environmental conditions, moisture retention, and material interactions. Identifying these high-risk areas is crucial for effective maintenance and preservation strategies.

**Expansion joints** are among the most susceptible components, as they are directly exposed to moisture infiltration and de-icing salts. These joints often serve as entry points for corrosive substances, accelerating deterioration in adjacent structural elements.

**Girder ends** are another critical area prone to corrosion. In modern weathering steel bridges, these sections are often painted to enhance durability [45]. However, the extent of the painted region varies significantly, ranging from a few inches to several feet. To maximize protection, it is recommended that the ends of girders be painted along a length equal to the girder’s depth.

**Bottom flanges of overpasses** are highly vulnerable due to their exposure to splash zones from vehicle lanes passing underneath, particularly in regions such as Ontario, where de-icing salts are frequently used [42]. These areas experience persistent wet–dry cycling, leading to accelerated corrosion.

Other bridge components susceptible to corrosion include:

- **Internal girders**, which are shielded from direct rainfall but can trap moisture, creating localized corrosion sites.
- **Interior surfaces of webs**, particularly in areas with insufficient drainage, where water can accumulate and promote corrosion.
- **Horizontal surfaces**, which tend to retain moisture and contaminants, increasing the risk of corrosion over time.
- **Sheltered conditions**, where reduced airflow prevents rapid drying, leading to prolonged moisture exposure and corrosion initiation.
- **Bolted joints**, which are particularly prone to “pack rust” due to crevice formation and differential aeration effects.
- **Drainage systems**, where accumulated debris and standing water can cause corrosion of surrounding structural components.
- **The interior of box sections**, which can trap humidity and pollutants, accelerating hidden corrosion.
- **Concrete–steel interfaces**, where moisture ingress and Cl penetration can initiate corrosion beneath protective coatings.
- **Ledges, angles, and channels**, which often accumulate debris and water, creating localized corrosion hotspots.
- **Wide flange shapes**, whose exposed surfaces are susceptible to corrosion in high-humidity environments.
- **Meeting points of dissimilar metals**, where galvanic corrosion can occur due to differences in electrochemical potential.

Understanding these vulnerable areas enables engineers and inspectors to implement targeted mitigation strategies, such as protective coatings, improved drainage, and regular maintenance, to extend the service life of weathering steel bridges.

## 6. Maintenance

To ensure the long-term durability of weathering steel bridges and their effective corrosion protection, a structured maintenance approach is necessary to address corrosion risks and environmental exposure. Based on inspection findings, the following maintenance measures are recommended:

### 6.1. Debris and Contaminant Removal

○ Loose debris should be cleared using compressed air or vacuum cleaning equipment to prevent moisture retention and localized corrosion.
○ Poorly adhering rust layers must be removed to enhance the stability of the protective patina.
○ High-pressure washing should be used to eliminate damp debris and corrosive substances, particularly in areas contaminated with salt, to mitigate Cl-induced corrosion.

### 6.2. Leak Detection and Drainage System Maintenance

○ Water infiltration sources should be identified and repaired, particularly near expansion joints, by observing water flow during rainy conditions.
○ Drainage systems and downpipes should be regularly cleaned to maintain proper water flow and prevent excessive moisture accumulation on steel surfaces.
○ Vegetation around the bridge should be removed to minimize organic debris accumulation, which can trap moisture and accelerate corrosion.
○ If existing drainage systems are inadequate, new systems should be installed to redirect water away from both the superstructure and substructure.

### 6.3. Structural Protection and Sealing

○ Bolted joint crevices should be sealed to prevent ‘pack-out,’ particularly at the ends of weathering steel girders and end diaphragms, where moisture can accumulate and accelerate corrosion.
○ To prevent water ingress, substructures should be wrapped in protective sheeting, such as polyethylene, until deck and runoff diversion systems are fully implemented [31,46].

### 6.4. Surface Treatment and Corrosion Mitigation

○ Rust stains on concrete piers and abutments should be removed using water blasting, sandblasting, or chemical stain removers. Additional protective measures, such as installing drip pans and plates, should be considered to prevent recurring staining [47].
○ Severe and progressive corrosion may require an acid solution wash to restore steel surfaces [45].
○ Weld spatter, rust deposits, coatings, foreign matter, oil, grease, and cutting residues should be properly removed to maintain surface integrity [31].
○ Post-construction stain removal methods, including water blasting, abrasive cleaning, or proprietary chemical stain removers, should be employed to minimize staining and enhance surface protection.

By implementing these maintenance strategies, bridge inspectors and engineers can effectively mitigate corrosion risks, enhance the performance of weathering steel structures, and extend service life in challenging environmental conditions.

It is recommended to regularly replace seals, drainage components, and joints as part of routine maintenance, rather than waiting for issues to arise. If a weathering steel structure fails to meet performance standards due to environmental conditions or design issues, supplementary corrosion protection coatings should be considered. Areas beneath deck joints, around less effective gaps, and girder ends with inadequate drainage may benefit from these coatings, but they should be applied only after thoroughly cleaning existing rust and contaminants. Wet abrasive blasting is suggested for removing pits caused by corrosion, especially when exposed to chlorides [48]. Risk-control points (RCPs) can be employed to minimize maintenance costs during planning, design, and fabrication by taking into account the degradation of member materials or during service by monitoring [48].

**Figure 10:**
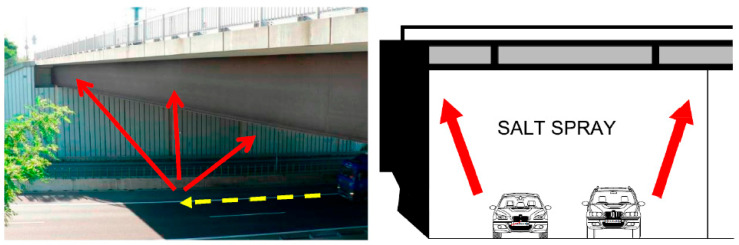
Splash zone in a tunnel effect environment [50].

During maintenance, it is crucial to remove the de-icing agent to prevent corrosion. Even after the final de-icing application, higher deposition rates can still be measured for up to two and a half months. This extended exposure period can lead to severe damage, especially if a tunnel effect occurs. Figure 10 illustrates the splash zone in a tunnel effect environment. The high concentration of dust deposits, including chlorides, has a notably adverse impact, particularly on the non-ventilated inner surfaces of the bottom flanges, where it hinders the effective formation of a protective corrosion layer. The motion of vehicles generates air turbulence, potentially causing air to splash onto the bridge girders. Traffic volumes, vehicle speeds, and the proportion of trucks all influence the distribution of road salt particles and the splash zone areas. In locations where girders are located close to mountain slopes or are parallel to each other, local humidity tends to be higher, air circulation is limited, and steel surfaces remain exposed to moisture for longer durations. Additionally, Cl tends to accumulate in rust on girders that receive less natural cleansing from rain, making these areas more susceptible to severe corrosion when de-icing agents are used [31,40,49,50,51].

### 6.5. Graffiti Removal

The removal of graffiti from weathering steel bridges is a challenging task, which has prompted the consideration of strategies to prevent public access to the girders. However, it is essential to balance this goal with the need for access for inspection, surveillance, and cleaning purposes. Markings should also be removed from the weathering steel’s surface [46].

The following methods can be applied for the removal of graffiti [31,33,36,39]:

- Apply an anti-graffiti coating to the painted areas on the bridge, typically around the abutments. Keep in mind that this may hinder patina formation in those specific regions.
- Use a citrus-based cleaner on graffiti, preferably within 24 h of application, before it sets. Then clean it with low-pressure water at 4000 psi.
- Completely remove graffiti and the underlying protective patina layer through high-pressure water jetting at 10,000 psi. This approach will also remove the patina layer, resulting in a patchy appearance until it redevelops.
- Explore the use of dry ice for graffiti removal.
- If the graffiti is not objectionable, consider leaving it, as it will eventually blend into the patina as it naturally forms (the ‘do nothing’ approach).

### 6.6. Washing and Cleaning

Washing and cleaning of bridge members are essential aspects of bridge maintenance and conservation [31,33,36]. These methods can typically be categorized into two groups: dry methods, such as cleaning, and wet methods, like washing. Washing aims to eliminate chlorides, and when prioritizing, it is advisable to start with bridge members that have the highest Cl levels. Dry methods encompass sweeping, compressed air blowing, vacuuming, shoveling, brushing, scraping, other mechanical cleaning techniques, and vegetation removal. Wet methods include flushing, low-pressure washing (under 1000 psi), and high-pressure washing (between 1200 and 6000 psi). In wet methods, it is significant to maintain the spray nozzle at the same level as or above the target area, keep it within a practical range from the target area (typically 1 to 5 feet), and ensure the nozzle has a spray angle ranging from 0 to 15 degrees.

A 0° nozzle has proven more effective at removing loose rust flakes from a moderately deteriorated patina to expose the protective layer. However, it may not work as well on the top surface of girder bottom flanges with significant patina delamination and poultice corrosion, where a portion of the deteriorated patina may remain and require higher pressure for better removal. On the other hand, the 40° nozzle easily covers the area but is inadequate for rust flake removal at a 3500-psi wash pressure [40]. It is recommended that the following members be thoroughly cleaned:

- Decks: Include roadways, expansion joints, drainage components, sidewalks, curbs, and railings.
- Superstructure: Focus on horizontal surfaces prone to debris accumulation, areas beneath deck expansion joints, and parts exposed to road-level splash.
- Substructure: Address abutment and pier seats, as well as splash-prone areas.
- Additional areas may need cleaning based on environmental factors:
- Superstructure: Cover entire girder lengths, diaphragms, and cross frames.
- Substructure: Attend to all exposed surfaces, especially those at risk of staining.

The frequency of cleaning and washing can be determined based on environmental conditions, traffic patterns, and moisture levels. It is worth noting that frequent natural washing, such as rainfall on a fascia girder, helps prevent Cl accumulation on weathering steel surfaces [36,40]. Although high-pressure washing may not be highly effective in removing chlorides penetrated within the patina, a study has suggested that periodic water washing could potentially reduce the corrosion rate [40].

### 6.7. Rehabilitation

In cases of severe corrosion, several methods can be used for rehabilitation, including crevice sealing, removal of poorly formed patina, and coating corroded weathering steel. It is also possible, in exceptional circumstances, to enclose the entire structure, although this is normally only feasible under exceptional circumstances. When considering painting as a rehabilitation method, the following steps should be taken:

- Dry blast all surfaces, if necessary, as obtaining a high-quality finish can be challenging due to the rough surface and pitting.
- Select a paint system that can accommodate significant variations in dry film thickness due to the rough steel substrate. Achieving a polished surface appearance may require up to four times the usual amount of primer used in a standard application.
- Ensure the chosen primer system resists the formation of rust residues and chemical residues, which can be challenging to completely remove from numerous pits in the substrate.
- Choose a paint system with a low water vapor transmission rate to prevent blistering [2,5,31,33,36].
- For connections that are not crucial to the structural integrity of the bridge, such as specific cross frame and lateral bracing members, it is permissible to disassemble the connection, perform dry blasting cleaning for the required surface preparation standard, apply a suitable coating, and then reassemble it. However, before disassembling any connection, a structural stability analysis should be conducted, and a disassembly and reassembly sequence should be formulated.
- In the case of vital structural connections, such as girder splices, the procedure involves using a penetrating sealer to displace moisture, sealing all edges with a compatible sealant (e.g., epoxy), and applying a compatible coating as a stripe coat to the connection [2,5,31,33,36].
- For corroded weathering steel in severe environments, suitable coatings include IOZ Epoxy, Polyurethane, Organic Zinc, Epoxy, and Polyurethane, as well as Thermally Sprayed Zinc/Epoxy. In mild/moderate environments, MCU Zinc, Epoxy, Polyurethane, epoxy mastic, and polyurethane are recommended. Applying a 3-layer coating on new weathering steel is beneficial, as it allows for better coating maintenance due to improved surface preparation, the absence of salt contamination, and potentially better coating application [52].

## 7. Design and Construction

### 7.1. Recommendations

The uniformity of the patina layer is a critical factor in corrosion protection performance of the weathering steels, as well as its visual appearance. Design and construction of the bridge have an important impact on the formation of the uniform patina layer. To achieve uniformity of color on the entire exterior face of the girder, it is necessary to lightly sandblast and weather it, especially if there are stains [43,44]. Pianca (1994) [53] reported that blast-cleaned samples initially perform better than mill scale, providing uniformity and accelerating patination. Blast cleaning not only provides a uniform surface but also accelerates the patination process. However, after five years, research shows no significant performance difference between sandblasted and mill scale surfaces [54,55]. The following design and details can be considered in the design of WS bridges [56,57]:

- Thin flange widths.
- Splice plates with a narrow profile to prevent ponding water at the leading edge.
- Stiffeners that do not trap water.
- Use of a minimum number of transverse and longitudinal stiffeners.
- Installation of water diverter plates on the bottom flanges.
- Painting the ends of steel members below joints, including integral abutments.
- Reducing the use of scuppers.
- Elimination of lateral bracing at the bottom of the flange, even on curved girders.
- To prevent water from flowing over or along the steel components, expansion joints should be positioned at a distance from the structural steel elements.
- To prevent water from running along the underside of the flange, it is advisable to extend the web plates of box girders approximately 20 mm beyond the bottom flange, ensuring that water flowing down the web drips off.
- The presence of a horizontal drip channel on the underside of the concrete deck at the joints seemed to effectively divert water from the girder ends [40].
- Minimizing or eliminating joints.
- Inclining horizontal surfaces.
- Avoiding corners that face inward.
- Applying seals to box sections.
- Incorporating diversion plates or weep holes for efficient water drainage.
- Using blast to clean mill scales on all girders.

According to the British Columbia Construction Manual [58,59], weathering steel structures require a specific coating system. This coating should cover all structural steel elements, except surfaces in contact with concrete, such as those involved in bolted connections, diaphragms, and bracing. The chosen coating system should be selected from the Ministry’s Approved Products List (AMS-STD-595A [60]). In BC, the coating must extend from deck joints (expansion and fixed) by the greater of 3000 mm or 1.5 times the structure’s depth. In areas where exposure to marine conditions or de-icing agents is a concern, applying a protective coating to the steel is essential. The designer must incorporate design features that prevent water accumulation on girder flanges. In BC, it is advised that in areas where water pooling cannot be avoided, an immersion-grade coating is required [58,59]. In Ontario, weathering steel girders should have a coating extending 3 m from their ends, apart from the area beneath expansion joints [61,62]. Texas Quality Steel Council guidelines [46], require additional cleaning (solvent, hand, power brush, or blasting; no acids) after welding and concrete placement.

According to U.S. guidelines [31], I-girder bridges typically perform best when their decks are continuous, often incorporating composite reinforced concrete decks. On the other hand, box girder bridges tend to exhibit superior performance, provided that the interiors of the box sections remain dry. This enhanced performance can be attributed to the absence of extensive external horizontal surfaces where water and salts can accumulate. It is also important to secure drainage holes with screens to prevent wildlife from nesting within enclosed boxes. In addition to box girders, bridges may include closed sections, such as boxes used for various structural elements. To prevent accelerated corrosion due to potential water accumulation, it is advisable to apply a minimum of one coat of a light-colored primer to the interior surfaces of these built-up members.

In areas where the level of corrosion is uncertain or confirmed to be high, it is advisable to consider adding an additional protective layer to ensure adequate structural strength. Sacrificial thickness is often a more economical long-term solution than future maintenance painting, especially when long-term performance is uncertain. Assuming a high corrosion rate of 0.0008 inches/year (equal to 20.32 µm/year), a corrosion allowance of 1/16 inch should be sufficient to maintain the necessary thickness over a 75-year service life. Extending the service life to 100 years is estimated to result in an additional thickness reduction of less than 0.02 inches compared to the original thickness without the corrosion allowance, resulting in a total decrease of approximately 0.08 inches [31]. For reference, Table 5 provides corrosion allowance values for European countries and Australia [33,36].

**Figure 11:**
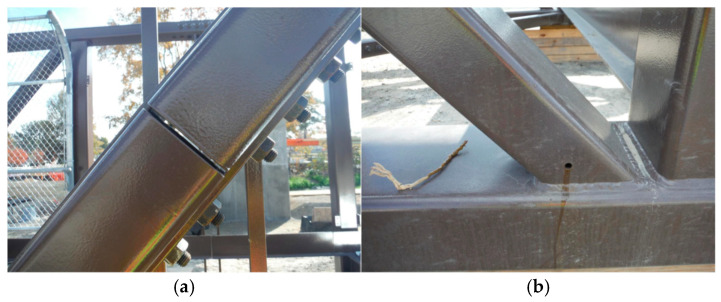
Example of a poorly detailed HSS pedestrian bridge: (**a**) splices allow moisture to enter the inside of closed sections; (**b**) drain holes are placed too high on the vertical and diagonal members, allowing water to pond on the inside below the drain holes [31].

The recommended practice for deck types in WS bridge construction involves using decks that provide water-tight protection for superstructure components, such as reinforced-concrete decks. Timber or open steel grid decks should be avoided due to water ingress and continuous wetness. If timber is necessary, use mastic strips or damp-proof materials between the deck and girders to mitigate corrosion. Furthermore, it is advisable to maintain adequate girder spacing, preferably around 6 feet or less than the girder depth. This promotes drying, prevents debris buildup, and facilitates inspections. However, closer spacings can be used for railroad structures with limited exposure to chlorides [31]. The design of stiffeners should prioritize effective drainage. Effective drainage in stiffeners can be achieved through enclosed triangular or trapezoidal shapes, or by incorporating drainage passages with at least a 50 mm radius (see Figure 11).

To minimize direct rainwater exposure, it is recommended to ensure that the projection of the bridge deck extends to a height equal to or greater than that of the steel girder. In the case of box sections, the lower flanges should not extend horizontally; instead, extended web plates beneath their respective lower flanges, encompassing all welds, can create drip features. For the outlet pipes at the abutment, it is essential to ensure they are long enough to prevent water discharge from splashing onto nearby steel elements or the substructure, regardless of wind conditions. Furthermore, it is advisable to prefer drainage components made of non-metallic materials [31,33,36].

### 7.2. Things to Avoid in the Design

Design recommendations prioritize specific factors aimed at minimizing the presence of stagnant water on steel surfaces. These factors, as suggested by previous studies [39,40,56], include:

- Reducing or eliminating joints by utilizing integral abutments.
- Incorporating horizontal surfaces with a slope.
- Minimizing the risk of re-entry.
- Sealing the sections of the box.
- Designing diversion plates or weeps to facilitate water drainage.
- Ensuring that stiffeners not welded to the bottom flange extend at least 30 mm above the flange.

In addition to these factors, various elements such as shelters, orientation, angle of exposure, time of wetness, airborne exposure, pollutants, debris, moisture effects, and the geometric details of structures can impact microclimates [40].

### 7.3. Conditions Not Recommended for Using WSs

There are a number of environments where the application of WSs is not recommended. Those include:

- Harsh environments, such as marine settings with high Cl exposure.
- Structures or structural elements that may accumulate significant Cl deposits due to de-icing salt usage.
- Continuous exposure to wet or damp conditions can affect steel.
- Buried steel in soil should be protected from corrosion by applying appropriate coating. However, steel structures embedded in concrete, with sufficient concrete cover, typically do not require a protective coating for longevity.
- In environments with extreme atmospheric pollution or concentrated corrosive industrial fumes, although this is less of a concern today.
- Bridge crossings over bodies of water with limited headroom, such as 2.5 m in Ireland.

It is worth noting that the weathering steel has demonstrated satisfactory performance in many river bridges. In cases where tree foliage or other obstructions hinder natural drying, steel members may remain consistently wet or damp. Interestingly, weathering steels are less susceptible to corrosion in stagnant water compared to open-air exposure [63]. This phenomenon can be attributed to the increase in stagnant water pH over time, resulting from low oxygen levels and a high concentration of FeOOH. Notably, the chromium content in the outer rust layers surpasses that found in the inner rust layers. A study conducted after 30 years has indicated that weathering steels can be used for guardrails if the component geometry is selected to prevent rainwater accumulation, thus avoiding a wet surface on the steel [64].

## 8. Future

While weathering steels inherently resist corrosion through the formation of a protective patina, ongoing research and development efforts are dedicated to expanding their performance envelope and addressing existing limitations. These future developments are geared towards enhancing their suitability for a wider array of environments, refining their esthetic qualities, and broadening their spectrum of applications.

A significant area of focus is the creation of high-performance weathering steels. This involves the development of novel alloys exhibiting superior resistance to aggressive conditions, particularly those characterized by high salinity, such as coastal regions, and significant levels of industrial pollution. As mentioned by Phuong & Bach (2008) [25], the presence of elements like silicon, chromium, nickel, copper, and phosphorus in the surface rust layers contributes to improved corrosion resistance in such atmospheres, with chromium and iron forming a stable, nanoparticle-sized α-(Fe_1−x_Cr_x_)OOH compound on the steel matrix. Achieving this enhanced corrosion resistance often necessitates precise adjustments to the steel’s chemical composition, including increased proportions of elements like nickel and other intentional alloying additions [65], to foster the development of a more stable and impermeable patina even in challenging exposures. Such advancements would enable the design of more efficient and small structural elements, thereby contributing to reduced material consumption and a lower embodied carbon footprint. The development of high-strength weathering steel grades, with yield strengths already reaching up to 960 MPa [66], exemplifies this trend. The establishment of industry standards and performance-based guidelines specifically for these high-performance weathering steels will be crucial to ensure their reliable long-term durability and dependability in demanding applications. Modified alloying concepts, potentially informed by computer simulations, are also being suggested to optimize weathering steel standards [67].

Addressing the initial esthetic concerns associated with the early stages of rust formation and the potential for staining adjacent materials is another key area of development. Advancements in pre-weathering technologies are crucial in this regard. These techniques involve carefully controlled industrial processes, such as exposure to oxidizing agents and controlled cycles of wetting and drying, to cultivate a uniform and stable patina on the steel surface prior to its installation [68]. Future efforts in this domain are likely to prioritize the development of more sustainable pre-weathering methods, minimizing the use of harsh chemicals and ensuring responsible waste management. Furthermore, research may explore innovative approaches to precisely control the final color and texture of the pre-weathered patina, satisfying the specific esthetic demands of diverse architectural projects.

The surface treatments also present opportunities for future development. The creation of clear, durable sealants that can be applied to a mature patina to effectively prevent further rust runoff and staining of surrounding materials is an area of ongoing interest [69]. These sealers aim to preserve the desired esthetic while modifying potential drawbacks.

The integration of weathering steel with advanced manufacturing and digital technologies holds significant promise. Techniques such as robotic welding and potentially additive manufacturing could facilitate the creation of more complex and intricate weathering steel structures with enhanced precision and efficiency. Moreover, the incorporation of weathering steel’s unique properties into Building Information Modeling (BIM) and sophisticated structural analysis software will enable the design of more optimized structures, taking into account long-term corrosion behavior and environmental influences [69]. The exploration of artificial intelligence (AI) and machine learning for the structural health monitoring of weathering steel structures could provide valuable information on the weathering steel’s long-term performance and maintenance requirements.

In line with growing sustainability laws, the inherent durability and low maintenance demands of weathering steel strongly align with principles of environmental responsibility, minimizing the need for coatings and replacements throughout a structure’s lifespan. An exciting future prospect is the potential for fossil-free weathering steel production, which would further diminish its environmental footprint. Promoting the recyclability of weathering steel will also be a crucial aspect of its sustainable application within a circular economic framework.

The distinctive combination of structural performance and esthetic appeal is driving the expanding adoption of weathering steel across a diverse range of applications, including infrastructure projects, architectural applications, artistic expressions, transportation applications, and security solutions. By proactively addressing current limitations and capitalizing on the ongoing advancements in materials science, manufacturing, and digital technologies, weathering steel is poised to assume an increasingly prominent role in sustainable and esthetically driven construction and design in the years ahead. The development of new weathering steel concepts specifically for challenging environments like coastal areas, based on long-term exposure testing, further emphasizes this trend. 

## 9. Summary

A comprehensive review of the performance of weathering steel (WS) bridges was provided, with a particular focus on their behavior in Canadian climates. The review delves into the fundamental aspects of the mechanism by which the weathering steel achieves its corrosion resistance through the formation of a protective patina, the factors that influence this process, and the essential strategies for long-term maintenance.

The main advantage of weathering steel lies in its ability to form a stable, adherent rust layer, known as a patina, when exposed to the atmosphere. This patina, primarily composed of stable iron oxyhydroxides, acts as a natural barrier against further corrosion, eliminating the need for painting, which results in reducing maintenance costs and increasing durability. The main alloying elements within the chemical composition of WS were also discussed, highlighting the crucial roles of alloying elements such as copper, chromium, nickel, and niobium in promoting the formation and stability of this protective layer.

However, the effectiveness of the patina is highly dependent on environmental conditions and proper bridge design. It was emphasized that factors like appropriate wet–dry cycles are necessary for patina development, while aggressive elements such as chlorides (from de-icing salts or marine environments) and sulfur dioxide (from industrial pollution) can disrupt the patina and accelerate corrosion. Prolonged periods of wetness are particularly detrimental.

Microenvironments within a bridge structure, such as areas prone to debris accumulation, inadequate drainage, or sheltered locations that remain damp, are identified as critical areas where accelerated corrosion can occur. The importance of thoughtful bridge design to minimize these vulnerabilities is recognized, and the need for features that ensure good drainage and avoid moisture traps.

Regular inspection and proactive maintenance are presented as indispensable for ensuring the long-term performance of WS bridges. This includes routine checks for debris and salt accumulation, inspection of drainage systems and expansion joints, and targeted cleaning to remove corrosive substances. Visual assessment of the patina’s appearance (color, texture, and adherence) is a key part of inspections, although it is recognized that visual cues can sometimes be misleading, necessitating more detailed evaluations like tape tests and ultrasonic measurements.

The impact of climate change on the corrosion performance of WS bridges was also considered. Rising global temperatures, altered precipitation patterns, and phenomena like ocean acidification are projected to increase the aggressiveness of environments, potentially accelerating corrosion rates and reducing the service life of existing structures, particularly in coastal regions.

Lastly, the future directions in weathering steel research and application are discussed. This includes the development of new, high-performance alloys with enhanced resistance to aggressive environments, advancements in pre-weathering techniques for improved initial esthetics, and the exploration of protective sealants and coatings for vulnerable areas. The integration of digital technologies, such as BIM and AI for design optimization and structural health monitoring, is also highlighted as a promising area.

## Figures and Tables

**Table 1:** The chemical compositions of WS in ASTM A-242 and ASTM A-588.

WS	C	Mn	P	S	Si	Cu	Ni	Cr	V	Al
ASTM A-242	0.11	0.31	0.092	0.02	0.42	0.30	0.31	0.82	<0.01	0.08
ASTM A-588	0.13	1.03	0.006	0.019	0.25	0.33	0.015	0.56	0.038	0.043

**Table 2:** Most commonly used weathering steels around the world (US: United States of America, UK: United Kingdom, EU: European Union, AU: Australia) and a comparison of their designations.

US	UK/EU	Japan	China	AU	Key Features/Applications
ASTM A-242 (Type 1)	EN S355J0WP [10]	JIS SPA-H [11]	GB/T Q235NH [12]		Original COR-TEN A, thinner plates, housing, freight cars, architectural. Higher phosphorus.
ASTM A-242 (Type 2/Corten B)	EN S355J2W [10]	JIS SPA-C [11]	GB/T Q345NH [12]		Thinner plates, urban furnishing, passenger ships, cranes. Lower phosphorus.
ASTM A-588 (Grade A)	EN S355J2W [10]	JIS SPA-C [11]	GB/T Q345NH [12]	AS/NZS 3678 WR350A (L0, L20) [13]	Higher strength, welded bridges, structural shapes, plates, bars. Higher phosphorus (Type A Aus).
ASTM A-588 (Grade B)	EN S355J2W [10]	JIS SPA-C [11]	GB/T Q345NH [12]	AS/NZS 3678 WR350B (L0, L20) [13]	Higher strength, welded bridges, structural shapes, plates, bars. Lower phosphorus (Type B Aus), better weldability.
ASTM A-588 (Grade C)					
ASTM A-588 (Grade K)					
ASTM A-606 [14]					Thin sheet, strip, coil, structural and miscellaneous purposes, enhanced corrosion resistance.
ASTM A709-50W [15]					Structural steel in bridges, high strength, low alloy, high corrosion resistance index.
ASTM A-847 [16]					Minimum yield 50 ksi, tensile 70 ksi. Limited information.
ASTM A871-65 [17]					High-strength plate, transmission and lighting poles, esthetic alternative to galvanized.
	EN S355J0W [10]				Equivalent to Corten B, impact test 0 °C.
	EN S355K2W [10]				Similarly to S355J2W, higher impact toughness at −20 °C.
	EN S355J4W [10]				Very low-temp applications, impact test −40 °C.
	EN S355J5W [10]				Extremely low-temp applications, impact test −50 °C.
	EN S235J0W/J2W [10]				Lower strength grades.
	EN S420J2W/S460J2W [10]				Higher strength grades, used in bridges.
	PATINAX 355P (S355J2WP+N) [10]				Equivalent to Corten A, higher phosphorus, higher corrosion resistance.
	PATINAX 355 (S355J2W+N) [10]				Equivalent to Corten B.
	PATINAX 275PK (S355J2WP) [10]				Thinner cold-rolled sheet, higher phosphorus.
		JIS G3114 SMA400AW [18]			Weldable, no low-temp impact test requirement.
		JIS G3114 SMA400BW [18]			Weldable, impact test 0 °C min 27 J.
		JIS G3114 SMA400CW [18]			Weldable, impact test at lower temp than BW.
			GB/T Q295NH [12]		Welding weathering steel.
			GB/T Q355NH [12]		High tensile strength, good weldability, low-temp impact toughness.
			GB/T Q460NH/Q500NH/Q550NH [12]		Higher strength welding weathering steels.
			GB/T Q265GNH/Q295GNH/Q310GNH/Q355GNH [12]		High weathering steel grades.
				AS/NZS 1594 HW350A [19]	Hot-rolled flat product, good formability.
				AS/NZS 1595 CW300A [20]	Cold-rolled, smoother finish, tighter tolerances.

**Table 3:** The category of time of wetness according to the US guidelines.

Category	Time of Wetness (h/Year)
T0	NA
T1	10
T2	250
T3	2500
T4	5500
T5	>5500

**Table 4:** Relationship between rust layer thickness and appearance [38].

Condition	Rating	Appearance	Thickness
Normal	5	The tone is bright, and the color is orange and yellow brown. There are almost no bumps, and the rust particles are very small. The amount of rust is little, and the maximum particle size is less than 1 mm.	Below 200 µm
Normal	4	The tone is dark brown, and the color is uneven. There are almost no bumps, and the rust particles are fine and uniform. The amount of rust is more, and the maximum particle size is less than 1 mm.	Below 400 µm
Normal	3	The tone is dark brown to brown, and the color is uneven. There are slight bumps, and the rust particles are uneven. The amount of rust is large, and the maximum particle size is less than 1–5 mm.	Below 400 µm
Observation required	2	The tone is dark brown to brown, and the color is uneven. There are large bumps, and the rust particles are rough and scaly. The amount of rust is large, and the maximum particle size is less than 5–25 mm.	Above 200 µm–below 800 µm
Abnormal	1	There are various local tones. There are large bumps, and the rust layer occurs layered peeling.	Above 800 µm

**Table 5:** Corrosion allowance (mm) for the thickness loss in 100 years [33,36].

Country	Allowance				
	C1	C2	C3	C4	C5
Belgium	-	0.11–0.8	0.53–1.2	1.05–1.5	Not allowed
Germany	-	0.8	1.0	1.5	Not allowed
UK (120 years)	1.0	1.0	1.0	1.5	Not allowed
France	N applicable	1.0	1.0	1.5	Not allowed
Spain	1.0	1.0	1.0	Not allowed	Not allowed
ECCS	N applicable	0.8	1.0	1.5	Not allowed
Australia	1 (0.5 for internal)	1	1.5	Not allowed	Not allowed

## Data Availability

No new data were created or analyzed in this study. Data sharing is not applicable to this article.
